# Rise of Big Data in Spine Surgery Research: A Bibliometric Analysis, 1993-2024

**DOI:** 10.7759/cureus.99856

**Published:** 2025-12-22

**Authors:** Julius Gerstmeyer, Anna Gorbacheva, Clifford Pierre, Alexander Von Glinski, Tara Heffernan, Luke Jouppi, Periklis Godolias, Thomas A Schildhauer, Amir Abdul-Jabbar, Rod J Oskouian, Jens Chapman

**Affiliations:** 1 Department of Orthopedics and Trauma Surgery, Katholische Kliniken Bochum, Bochum, DEU; 2 Department of Orthopedics and Trauma Surgery, BG University Hospital Bergmannsheil, Bochum, DEU; 3 Department of Neurosurgery, Seattle Science Foundation, Seattle, USA; 4 Department of Neurosurgery, Swedish Neuroscience Institute, Seattle, USA; 5 Department of Orthopedics and Trauma Surgery, St. Josef Hospital Essen-Werden, Essen, DEU; 6 Department of Orthopedics and Spine Surgery, Swedish Neuroscience Institute, Seattle, USA

**Keywords:** bibliometric analysis, big data, large database, research, spine, spine surgery

## Abstract

Spine surgery represents a dynamic and innovative specialty with a steady increase in annual publications since 1900. The introduction of large databases and registries marked a paradigm shift in research. Despite their quantitative superiority, databases are inherently limited in a number of ways. Spine surgery remains a somewhat controversial specialty with significant cost, population health, and outcomes implications, and has, not surprisingly, become more frequently associated with large-scale registry studies. The objective of this study was to descriptively characterize trends in large-database usage in spine surgery research over time, including publication volume, topic distribution, and geographic output.

A database search in the Clarivate Analytics Web of Science (WoS) database with keywords (“big data” OR “large database” OR “registry” OR “national database”) AND “spine surgery” was conducted on July 7, 2025, and original and review articles published between 1993 and 2024 were included. Retrieved articles were categorized into seven areas based on primary research objectives, and data underwent descriptive quantitative analysis. Descriptive analyses were performed in Microsoft Excel (Microsoft® Corp., Redmond, WA, USA), and keyword co-occurrence network and overlay visualizations were generated using VOSviewer (Version 1.6.20, Centre for Science and Technology Studies, Leiden University, The Netherlands).

A total of 633 articles were analyzed, revealing a more than 10-fold increase in publications from 2012 to 2024. The degenerative category was most common (189 articles), followed by public health (179) and infection (75). The United States dominated the research output (62.29%). Raw annual citation counts also significantly increased, from 146 in 2012 to 2,132 in 2024.

The utilization of large databases in spine surgery research has substantially grown, reflecting a shift toward big data usage. Publications on spinal infections and public health outcomes have notably increased, while degenerative conditions consistently prevail. Geographic distribution remains skewed towards western countries, especially the United States. These trends suggest an expanding recognition of database studies as a critical tool for evidence-based research in spine surgery.

## Introduction and background

Spine surgery remains a complex and dynamic specialty, reflected in a constant increase in scientific research articles. Overall, around 35,500 spine surgery-related studies have been published since 1900, with a steady annual increase of around 2.8% [1]. The quest for greater scientific validation has led to an increasing effort to utilize ever larger pools of patient data, combined with more evolved analytical technologies, that have been merged into large-scale databases, also known as “BIG DATA”, a term first popularized by John Mashey at Silicon Graphics, Inc. in the mid-1990s [2].

Large datasets, or “Big Data”, used in research are commonly derived from administrative databases or registry-based systems. The primary goal of administrative databases is usually financial and infrastructurally targeted, and they are mainly maintained by institutional and third-party payer entities. Registries historically have been more specialty-driven initiatives, originated by physicians and researchers, and commonly represent voluntary not-for-profit efforts. The crucial governance of databases varies based on their stated purpose, and is structured and maintained by incorporated health systems, professional societies, governmental institutions, or, more recently, has also morphed into profit-oriented enterprises. In this era of evidence-based medicine, the availability of large datasets with capabilities to allow for epidemiologic research has revolutionized assessment of treatment impact by facilitating investigations into population health and outcomes, as well as allowing for improved risk-factor detection and big-picture trend analyses that were previously obscure [3-9]. Well-conceived large databases allow for enhanced retrospective research, with the benefit of analytical immediacy, as well as time- and cost-efficiency compared to traditional prospective and multicenter analyses. 

Inherent methodological limitations of scientific large-scale database usage include data reliability and validity, selection bias, incomplete data, and the application of normative categorizations while applying patient privacy statutes. Bias potential, in particular, is a very substantial threat to data validity, creating an onus on investigators to apply a realistic and transparent awareness of such influences [3,10,11]. The field of spine surgery can be considered one of the early adopters of database-derived research output, and has been described as the most frequent specialty utilizer [3]. The reasons for this may lie in the fact that the specialty of spine care, with its high clinical prevalence, remains mired in some controversy, perhaps due to its significant associated cost, high clinical prevalence, and questions pertaining to outcomes, and has, expectedly, become more frequently associated with large-scale data-gathering endeavors. 

Within the larger field of spine surgery, subspecialty publication variations and their dynamics over time have not been explored, and could help to improve our understanding of changing clinical concerns. We hypothesized that the proportional share of large-database publications would increase over time, especially in areas such as degenerative, deformity, and oncology-focused spine research. Our bibliometric analysis aims to provide a descriptive analysis and overview of the usage of large databases in various areas of spine surgery, including trends and changes in areas of focus, as well as their geographic representation.

## Review

Methods

A database search in the Clarivate Analytics Web of Science (WoS) Core Collection database was conducted on July 7, 2025. The Boolean search query included the keywords (“big data” OR “large database” OR “registry” OR “national database”) AND “spine surgery.” This search included titles, abstracts, author keywords, and Keywords Plus. The keyword set was selected to capture studies that explicitly describe the use of large databases or registries using generic, reproducible terminology. A full report was extracted, with only original and review articles included, while all other publication types were excluded. Data from 2025 were not included due to incomplete data for the year. The full report and citation analysis were exported.

All articles were categorized using a rule-based, semi-automated keyword approach based on their stated primary objectives, into the following domains: trauma; infection; degenerative disorders; deformity; oncology/neoplasia; public health/outcomes; and investigations with a primary focus on technology. The public health section included subdivisions into socio-economic, epidemiological, and non-specific patient-reported spinal surgery health and outcomes studies, while the technology sector was dedicated to non-specific surgical techniques and technologies, such as “minimally invasive surgery,” “artificial intelligence,” “machine learning,” and “natural language processing.” If multiple categories were matched, the article was assigned based on its primary stated objective, as described in the abstract.

A descriptive and quantitative analysis was performed using Excel (Microsoft®, Version 16.86, Redmond, WA, USA). For bibliometric network analysis of all-time data, including keyword co-occurrence and overlay visualizations, the bibliometric software VOSviewer (Version 1.6.20, Centre for Science and Technology Studies, Leiden University, The Netherlands) was used. All ranked analyses included the top 10 elements.

Results

Article Distribution and Publication Trends

After application of our search and filter modalities, a total of 633 articles were extracted from the Web of Science database during our investigational time frame from 1993 to 2024. Table 1 summarizes the yearly publications of each domain. The most frequent categories were degenerative disorders (189), followed by public health (179), infection (75), trauma (68), technology (68), deformity (35), and oncology/neoplasia (19). 

**Table 1:** Overview of yearly publications per category Categories reflect each article’s primary objective.

Publication Year	Trauma	Infection	Deformity	Degenerative	Tumor	Technology	Public health	Total
1993	-	-	-	1	-	-	-	1
2001	-	-	-	1	-	-	-	1
2005	1	-	-	1	-	-	-	2
2006	2	-	-	1	-	-	-	3
2007	-	-	-	1	-	1	1	3
2008	-	2	-	1	-	1	-	4
2009	3	-	-	-	-	-	-	3
2010	1	-	-	1	-	2	1	5
2011	-	-	-	-	-	1	1	2
2012	2	3	-	-	-	2	1	8
2013	2	1	-	1	-	5	5	14
2014	5	5	-	12	-	-	5	27
2015	1	9	-	15	-	1	10	36
2016	5	7	-	9	-	2	8	31
2017	4	8	2	11	-	5	15	45
2018	3	3	2	15	-	5	9	37
2019	3	4	9	25	1	3	14	59
2020	5	5	11	29	-	4	12	66
2021	5	4	8	17	7	7	20	68
2022	5	5	2	13	5	5	25	60
2023	10	12	-	17	4	13	29	85
2024	11	7	1	18	2	11	23	73
Total	68	75	35	189	19	68	179	633

**Figure 1:**
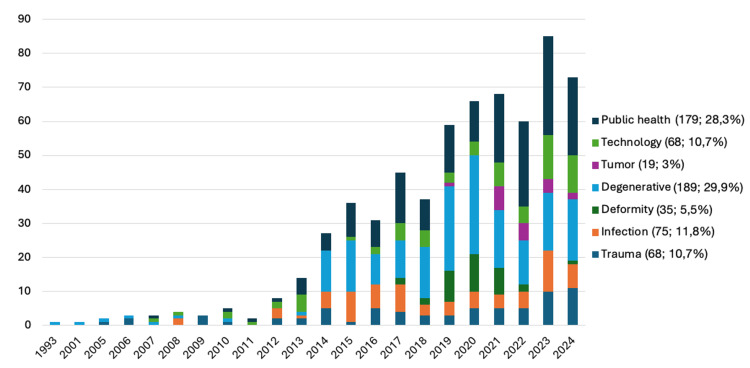
Overview of all publications (n = 633) per year and category Bar chart illustrating the number of publications per year across various categories from 1993 to 2024. Overall, the number of publications shows a general increase.

The overall number of database-derived publications demonstrated a substantial, greater-than-ten-fold increase from 2012 to 2023, with 8 to 85 publications (Figure 1). Categories such as trauma and public health exhibited consistent research output across multiple years, whereas infection, deformity, degenerative disorders, oncology/neoplasia, and technology showed greater variability in their publication frequency. Over the years, both public health and degenerative sections have seen the largest relative increase in publications per year, with a total of 23 and 18 in 2024, respectively. The most pronounced summative increase was found in the degenerative domain, starting with two publications in 2012 and ascending to 29 in 2023. 

Country Distribution 

**Figure 2:**
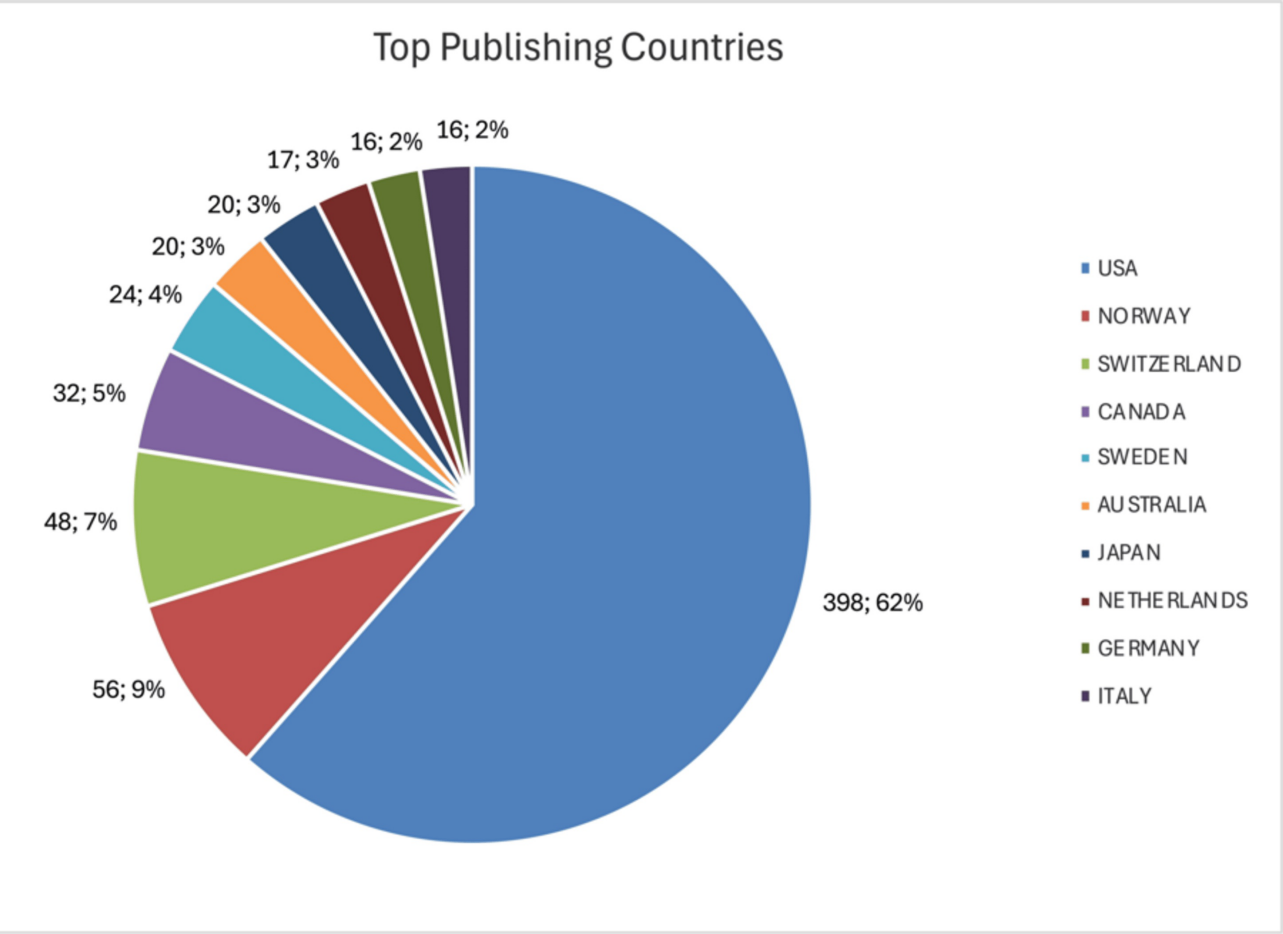
Distribution of 633 publications by country The pie chart displays the distribution of publications of the top 10 countries. The chart highlights a clear prominence of the United States, followed by Norway, Switzerland, and Canada.

The top publishing countries are shown in Figure 2. Our analysis showed a stark concentration of database research output, limited to a few countries. Authors affiliated with institutions in the United States of America published the most articles, accounting for a total of 62.29%. Norway and Switzerland followed, with 8.76% and 7.51%, respectively, while Canada and Sweden were represented with 5.01% and 3.76%. Further contributions, in sequence, ranged from Australia and Japan, both at 3.13%, down to the Netherlands at 2.66%. Sizeable population centers located in Asia and Africa were, however, not represented in the top 10 countries. These country shares represent absolute publication output and are not adjusted for population size, overall national research output, or database availability.

Raw Citation Analysis

**Figure 3:**
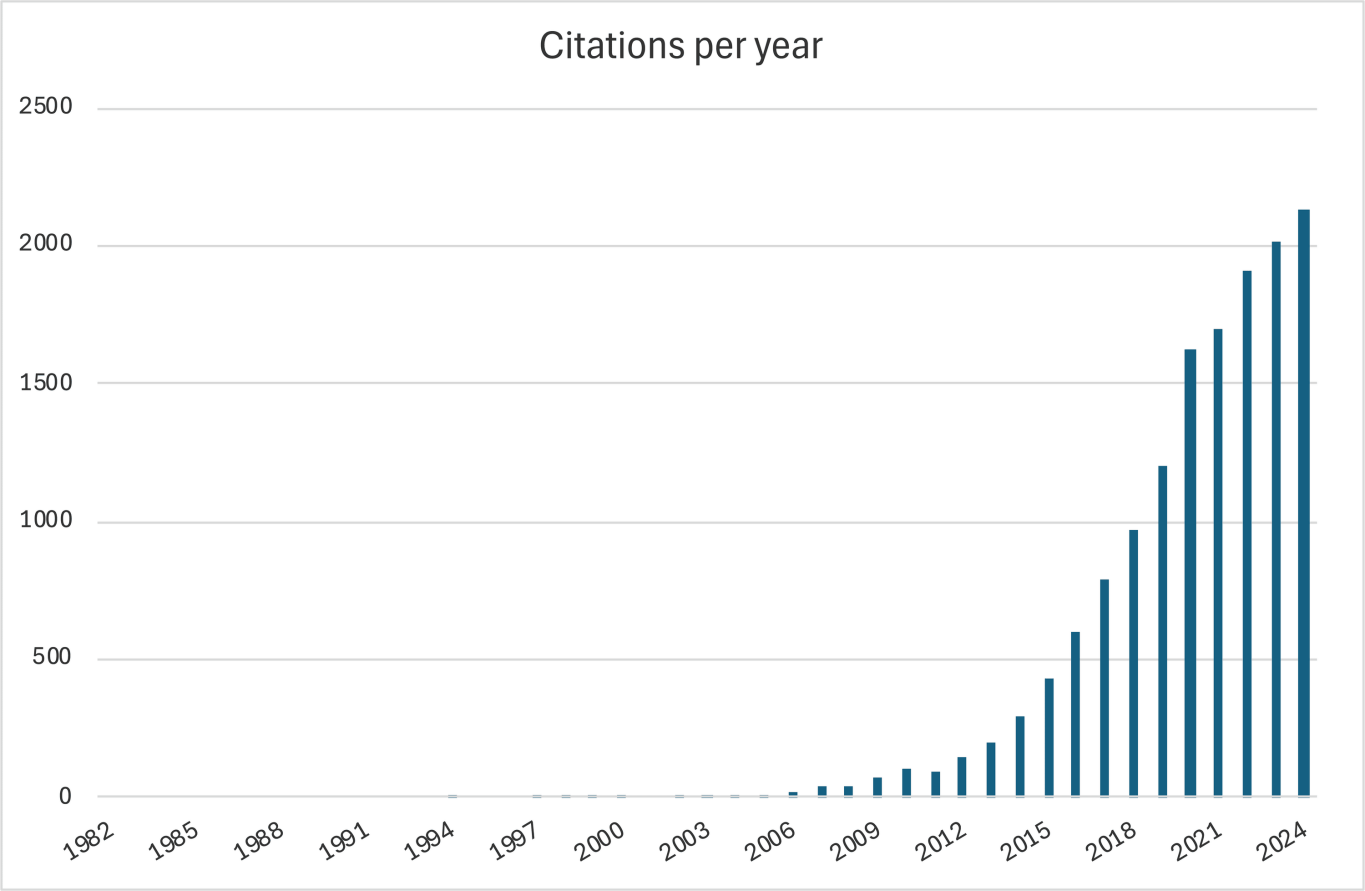
Raw citation analysis per year The bar chart shows the number of database-related raw citations per year from 2006 to 2024. A clear upward trend started in 2009, with a steady increase year over year.

Our citation analysis, as represented in Figure 3, shows a consistent increase in the number of raw citations of spine database studies from 2006 through 2024, with a particularly apparent year-over-year upward trend starting in 2009. Since 2016, with 600 citations, the greatest increase was seen in 2020, with 1,622 citations that year. 

Keyword Occurrence and Overlay Network Visualizations

**Figure 4:**
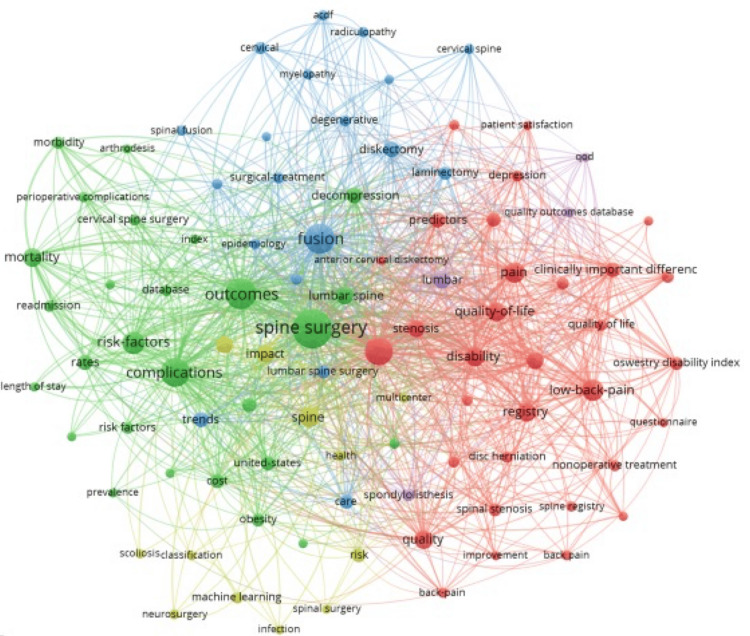
Keyword co-occurrence network visualization Each keyword is depicted as a node, with the size of each node representing the frequency of its use in the literature. Lines connecting the nodes represent their co-occurrence, as they appear together in individual publications. Thicker lines indicate stronger connections, or more frequent use of two words together. Each color represents a different theme.

Keyword co-occurrence mapping is displayed in Figure 4. Each keyword is depicted as a node, with the size of each node representing the frequency of its appearance in the published literature. Lines connecting the nodes represent their co-occurrence, as they appear together in individual publications. Thicker lines indicate stronger connections, or more frequent use of two words in combination with one another. Each color represents a different theme. Together, these elements provide a graphic representation of interconnectivity among topics. 

**Figure 5:**
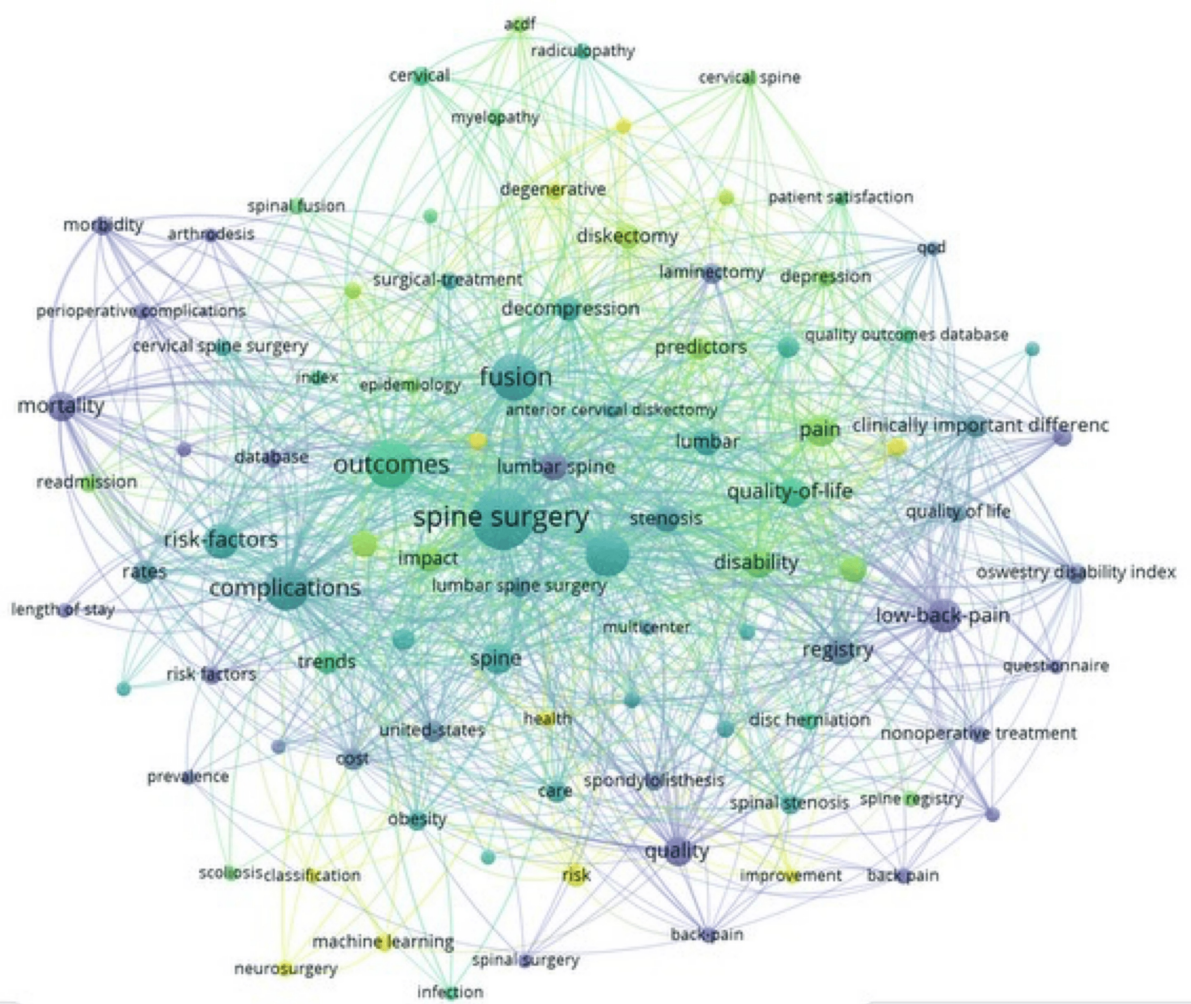
Keyword co-occurrence network visualization Each keyword is depicted as a node. The size of each node represents the frequency of its use in the literature. Lines connecting the nodes represent their co-occurrence, as they appear together in individual publications. Thicker lines represent stronger connections, or more frequent use of two words together. Each color represents a different theme.

In the overlay visualization (Figure 5), the term “machine learning” appeared in more recent years. The network visualization showed the most prevalent keywords to be “spine surgery”, followed by “outcomes”, “fusion”, and “complications.” Terms such as “quality of life”, “disability”, “questionnaire”, and “registry” appeared grouped together, while terms such as “risk factors”, “complications”, “length of stay”, “readmission”, and “database” occurred in close relation to one another. Other thematic clusters included words such as “machine learning”, “neurosurgery”, and “scoliosis”. Oncologic keywords did not appear in the analysis. 

Discussion

The emergence of large databases represents a true paradigm shift for medical research. Our bibliometric analysis provides a comprehensive overview of the utilization of large databases in spine surgery, highlighting trends, areas of focus, and their geographic distributions. The application of bibliometric analytics allows for a dynamic, time-based deeper understanding of research interests within the scientific community and accepted scientific principles [3]. However, given the descriptive nature of this bibliometric analysis, we present observed publication and citation patterns without implying causality where interpretation is offered.

Our analysis shows a greater-than-10-fold increase in total publications from 2012 to 2023, starting with eight publications and amounting to 85 more recently. Our finding is in keeping with other publications, such as Bohl et al., who showed that database utilization increased in orthopedic research overall, with its greatest usage in the subspecialty of spine surgery [3]. In contrast to the previous study by Kurland et al., who reported a steady 2.8% increase in total publications per year throughout spine surgery-related research, we found a far more dynamic and actually escalating database-derived research output [1]. This clearly underscores a growing value and interest in database analyses in spine research. 

Regardless of the specific increase in numbers, all of these findings, as well as ours, support the growing recognition of database-derived research as being meaningful in addressing complex clinical research hypotheses, such as outcomes and risk-factor analyses. In the analysis of such hypotheses, especially when utilizing large, heterogeneous, multi-center datasets and being cognizant of their potential limitations, these research tools have proven to be of increased validity in overcoming institutional bias. In general, the number of large database resources has increased logarithmically with rising computing power and enhanced end-user utility. While the exact number of available databases worldwide is unknown, noteworthy, sustained data collection efforts, such as The National Inpatient Sample (NIS) - an example of an administrative database implemented by the Healthcare Cost and Utilization Project (HCUP) - have been operational since 1988 [12]. In contrast, more focused databases and professional society-based registries, such as the Nationwide Readmissions Database (founded in 2010) and the Nationwide Ambulatory Surgery Sample (NASS, founded in 2016), have been operationalized more recently [13,14]. The crucial, increased focus on patient safety has been reflected in the creation of a targeted registry database in the form of the American College of Surgeons National Surgical Quality Improvement Program, established in 2005 [15]. The utility of such databases is shown by the fact that these are the most frequently used resources in orthopedic research [3,10]. Certainly, the increase in the number of available databases and the relative simplicity of performing large-scale population-based research may explain the increased representation in overall scientific research output. We observed higher publication counts during the 2019-2021 interval. Although this period overlaps with the COVID-19 pandemic, our data do not allow attribution of publication volume changes to pandemic-related disruptions or shifts in clinical activity. However, the benefits of accessing and analyzing large quantities of retrospective data in a contactless fashion might have been a factor contributing to that rise. We indeed found an increase in total publications during that time, from 59 in 2019 to 68 in 2021, dropping back down to 60 in 2022.

To our surprise, and running counter to our hypothesis, we found a substantial increase in database publications relative to spinal infections. This bibliometric analysis cannot determine the drivers of this trend. However, previous studies have reported contributing factors, such as a population shift toward a more multimorbid and aging population, which may increase susceptibility to infections. This may serve as an explanation for the increased prevalence of publications in this domain [16-18]. Furthermore, advanced imaging techniques have improved diagnostics of these conditions [19]. However, surgical site infections, as a sub-entity in the infection domain, remain a persistent challenge [20]. Thus, a growing recognition of spinal infectious entities, both the de novo and the perioperative variants, is starting to benefit from more structured, large-scale research in this area [21].

Overall, degenerative conditions expectedly remained the most frequent category of scientific publications in our analysis, as represented by a constant output over time. This trend can also be observed in the keyword network visualization (Figure 4), with spine surgery showing close interactions with degenerative- and outcome-related keywords. 

In contrast, despite a recognized increase in the incidence of oncologic diseases, the proportional scientific publication rate derived from large-scale databases has not, according to our bibliometric analysis, reached the degree one would expect [22,23]. In fact, oncologic topics remained the least frequent domain, totaling only one to seven publications per year, with 19 overall, despite a growing incidence affecting an increasingly aging population, and with well-recognized significant individual and societal impact, as well as associated cost of care [23]. A possible explanation could arise from the likelihood that multi-disciplinary specialties, such as oncology, may utilize databases and publication outlets beyond traditionally spine surgery-related publications. Again, this underrepresentation is supported by the keyword network visualization, which does not include oncologic keywords (Figure 4).

For public health- and outcomes-related research, we, however, found a constant and proportionally increasing output of publications, with many outcome-related keywords, such as “quality of life”, “complications”, and “satisfaction”, appearing in the keyword network visualization (Figures 1, 4). Large databases are particularly useful for such research, especially regarding social determinants of health, cost-effectiveness analysis, and patient-reported outcomes. 

The overlay visualization (Figure 5) indicates that “machine learning” appears predominantly in more recent years, and technology-category publications have increased over time. Previous bibliometric analyses of artificial intelligence in spine surgery and neurosurgery support this finding, with articles most notably discussing prediction modeling, as well as imaging and diagnostic aid [24,25]. 

The raw citation analysis revealed a constant increase, particularly from 2016 onwards, which was congruent with the increase in publications over time. This reflects a growing recognition and influence of database utility throughout the spine surgery research community. Additionally, it shows broad acceptance of large database analysis as a viable method for evidence-based research. Importantly, annual citation totals are influenced by time since publication and may, therefore, underrepresent the impact of the most recent years, and our counts were not adjusted for self-citation or normalized by output volume.

With the resources of large and controlled, federally funded administrative and patient care-based databases, the United States contributed the highest absolute output of scientific output in database research (62.29%). This might be due to the multitude of consistently maintained databases, with credible transparency and strictly enforced privacy rules, that represent ongoing investment in its academic endeavors [26,27]. Most other top 10 countries using databases, such as Norway, Switzerland, Canada, and Sweden, also represent high-income regions. This clear concentration of high-income countries illustrates the economic burden of maintaining large databases and supporting academic institutions [26,27]. This database utilization may, however, adversely influence the generalizability of results. Asia and Africa remain underrepresented in database research, despite their significant impact on global health, as cross-validation of database-derived insights to other regions of the world is currently limited. Interestingly, despite much of the bibliometric analytic software coming from the People's Republic of China, China itself is not represented in the top 10. However, because our analysis is not normalized by population, overall publication output, or database availability, these comparisons should be interpreted as output concentration, rather than per-capita or capacity-adjusted.

This bibliometric analysis is not without limitations. Data were solely acquired using the "Web of Science" database, potentially excluding studies indexed in other databases and limiting conclusions of the analysis to the output generated by the WoS search results. Further, the focus on generic keyword search terms may have undercaptured studies that used named databases as keywords but did not include generic terms such as "registry" or "national database" in the title, abstract, or keywords. The semi-automated categorization of articles, which was designed to be neutral in its application of selection criteria, may, by its very nature, introduce some inadvertent bias. Furthermore, some studies overlap with several domains and may not fit neatly into a single category, therefore leading to potential imprecision in classification and an oversimplification of complex, multi-dimensional research topics. Citation analyses were based on raw counts, were not normalized nor adjusted for self-citation, and are susceptible to citation lag, particularly for recent publication years. Overall, we found the application of bibliometric techniques to be demonstrative of the overall state of the field and its utilization of databases to date. 

## Conclusions

The use of large databases, also known as "BIG DATA", is showing an increasing impact on publications in spine surgery. The overall number of publications and citations has surged over the last decades, with a 10-fold increase from 2012 to 2023. The trend analysis showed increasing publications in infection and public health, whereas the degenerative category remained relatively constant. Oncology represented the smallest proportion, without a clear temporal increase. Our analysis reflects a growing acceptance of large database studies throughout spine surgery research. 
